# First Case of Monkeypox in Venezuela: Partial Complete Genome Sequence Allowed Its Grouping into the West African Clade II

**DOI:** 10.3390/tropicalmed8010002

**Published:** 2022-12-21

**Authors:** Pierina D’Angelo, Carmen L. Loureiro, Rossana C. Jaspe, Yoneira F. Sulbaran, Lieska Rodríguez, Víctor Alarcón, José Manuel García, José Luis Zambrano, Ferdinando Liprandi, Héctor R. Rangel, Flor H. Pujol

**Affiliations:** 1 Dirección de Diagnóstico y Vigilancia de Enfermedades Transmisibles, Instituto Nacional de Higiene “Rafael Rangel”, Caracas 1041, Venezuela; 2 Laboratorio de Virología Molecular, Centro de Microbiología y Biología Celular, Instituto Venezolano de Investigaciones Científicas, Caracas 1020, Venezuela; 3 Dirección General de Epidemiología, Ministerio del Poder Popular para la Salud, Caracas 1010, Venezuela; 4 Laboratorio de Virología Celular, Centro de Microbiología y Biología Celular, Instituto Venezolano de Investigaciones Científicas, Caracas 1020, Venezuela; 5 Laboratorio de Biología de Virus, Centro de Microbiología y Biología Celular, Instituto Venezolano de Investigaciones Científicas, Caracas 1020, Venezuela

**Keywords:** monkeypox, international outbreak, phylogenetic analysis, West African clade, clade II

## Abstract

The ongoing epidemic of monkeypox virus (MPXV) infection has already reached more than 50,000 persons worldwide until the end of August 2022. We report the first case detected in Venezuela. The patient reported traveling from Spain and contact with friends tested positive for MPXV after his return. Partial complete genome phylogenetic analysis allowed to group the isolate within the clade II of MPXV, the major one circulating worldwide. No other case of MPXV has been detected until the end of August 2022 in the country, although the presence of undiagnosed cases due to the fear of stigmatization cannot be ruled out.

## 1. Introduction

On 23 July 2022, the escalating global monkeypox virus (MPXV) 2022 outbreak was declared a Public Health Emergency of International Concern by WHO [1]. By the end of August 2022, more than 50,000 cases have been confirmed in almost 100 countries of the world [2,3].

MPXV belongs to the genus *Orthopoxvirus* of the family *Poxviridae*. The enveloped virion is around 250 nm × 360 nm. The viral genome is a double-stranded DNA of approximately 198 kb [4]. MPXV has an animal reservoir, probably rodents, and is endemic in countries from Central and West Africa, and infects humans through zoonotic events and then by human to human contacts [2]. 

Although MPXV infection is not documented as a sexually transmitted disease, it can spread through close contacts during sexual intercourse. The international outbreak has been characterized since May 2022, with a huge frequency of the cases among men who have sex with men, and this association had led to a fear of stigmatization [5].

**Figure 1:**
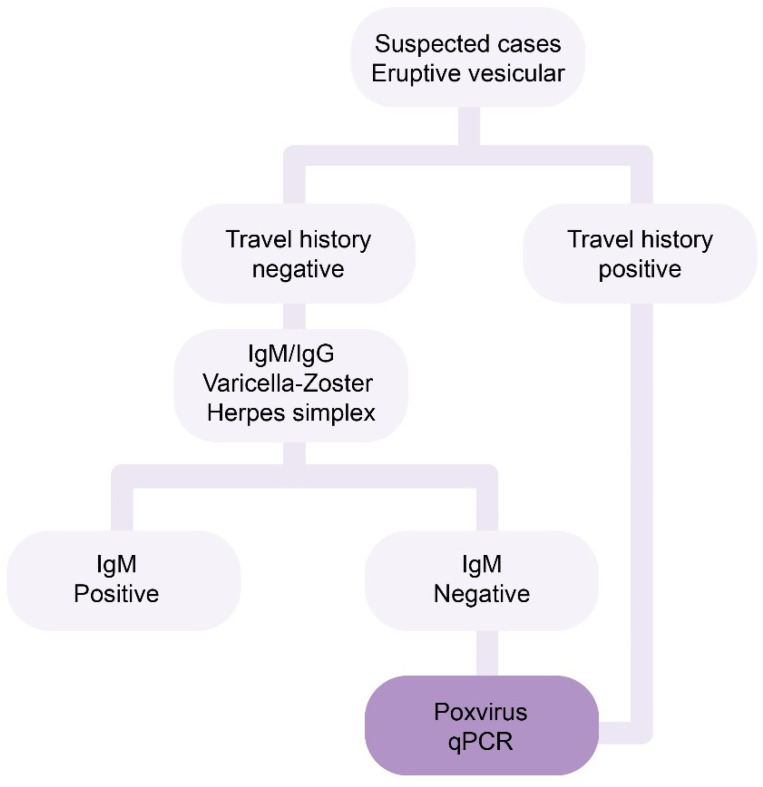
Algorithm from Venezuelan INHRR for the molecular detection of MPXV cases. Samples from patients with history of international travel were directly tested for the presence of MPXV DNA.

The Instituto Nacional de Higiene Rafael Rangel (INHRR) from Venezuela implemented an algorithm for the molecular detection of MPXV cases and serological discarding of other confusing exanthema-inducing infections (Figure 1). The aim of this study is to describe the first detection of MPXV infection in Venezuela.

## 2. Materials and Methods

For molecular detection, DNA was extracted from the swab of the skin lesions with the Vazyme DNA/RNA Extraction Kit (Shangai, China). Serum samples were also collected for the detection of IgM antibodies to Varicella (Varicella-Zoster Elisa IgG/IgM Vircell, Granada, Spain) and to Herpes simplex virus (Xema-Medica Co., Ltd., Moscow, Russia) (Figure 1).

The presence of MPXV DNA was detected by qPCR [6]. Other regions of the MPXV genome were also amplified [7,8] (Table 1). PCR-purified fragments were sent to Macrogen Sequencing Service (Macrogen, Seoul, Korea). Additionally, several libraries were prepared from the same sample, with 1, 5, 10, or 18 ng of total DNA, using the DNA Prep library preparation kit of the Nextera DNA CD Indexes (Illumina, Inc. San Diego, CA, USA), for next generation sequencing (NGS). The libraries were pooled and quantified (Qubit DNA HS, Thermo Scientific, Waltham, MA, USA). Their quality was checked (Bio-Fragment Analyzer, Qsep1-Lite, BiOptic, New Taipei city, Taiwan) before sequencing, using 10% PhiX control v3, using an iSeq 100 platform and a 300 cycle V2 kit with paired-end sequencing. The viral genome sequence assembly was performed using the Genome Detective Virus tool (https://www.genomedetective.com/) (https://www.genomedetective.com/ last accessed 10 September 2022) [9,10]. Nucleotide sequences of the partial complete genome have been deposited into the GISAID database with the accession ID EPI_ISL_15014548.

The FASTA file obtained from Genome Detective was analyzed using the Nextclade web-tool [11,12]. The JSON file containing the phylogenomic datasets was downloaded from Nextclade (https://clades.nextstrain.org/ last accessed 20 September 2022), and was analyzed at Auspice (https://auspice.us/ last accessed 20 September 2022) [13]. The alignment of MPXV genomes was performed using MAFFT v.7 (https://mafft.cbrc.jp/alignment/server/ last accessed 20 September 2022) [14,15]. Phylogeny reconstruction was performed using the IQ-TREE web-tool (http://www.iqtree.org/ last accessed 20 September 2022) with the GTR Model and 1000 ultrafast bootstrap repetitions [16,17]. Phylogenetic trees were visualized and edited with the iTOL (Interactive Tree Of Life) web-tool (https://itol.embl.de/ last accessed 20 September 2022) [18,19]. An EPS file was downloaded for final editing and formatting of the phylogenetic tree using Illustrator CS (ver. 23.0.1) [20].

## 3. Results

### 3.1. Case Report

One sample from the skin lesions of a patient was found positive for MPXV viral DNA by qPCR (Figure 1), with both the generic and the clade II (West African clade)-specific primers. The patient, who was a 32-year-old man, reported on May 28 the following symptoms: fever; headache; and cutaneous maculopapular and vesicular rash on the face, back, chest and arms. He also presented with generalized abdominal pain and diarrhea. He returned from Spain on 4 June 2022, and after several stopovers, arrived in Venezuela on 6 June 2022. He was then informed that the two friends he visited in Barcelona, Spain, were diagnosed with MPXV, with the condition unknown when he traveled. He flew on 7 June 2022, from the capital Caracas to his residence in Barinas state, where he kept isolated. He then called a physician, who contacted the service of the Epidemiological Surveillance Direction of his residence zone. Swabs from the lesions and nasal region were collected on June 10, and sent to INHRR. The patient was also found negative for COVID-19, antibodies against HIV, and IgM antibodies against Varicella-Zoster and Herpesvirus. On day 24 since the beginning of symptoms, the patient only harbored scars, without any other symptoms, and was allowed to suspend his isolation.

The international air flight companies were informed of the case. In Venezuela, 33/46 of the passengers who traveled with the patient in the national air flight were monitored for 63 days, as well as his two parents. None of them developed symptoms suggestive of MPXV infection.

### 3.2. Phylogenetic Analysis

**Figure 2:**
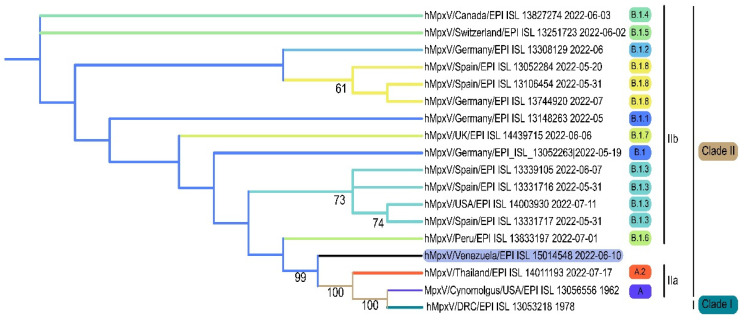
Phylogenetic analysis of the first case pf MPXV in Venezuela. The different lineages described for clade IIb are shown.

The sample was also positive by PCR targeting other regions of the viral genome: the hemagglutinin gene [8], and the ORF F4L and E5 [7]. The complete MPXV genome was assembled from several libraries processed by NGS, and sequences from the PCR-amplified products were also used for the final assembled sequence. A total of 85,382 nt were deciphered with the different strategies. The time of collection of the sample (13 days after the development of symptoms) may have hampered to obtain a higher coverage of the next generation sequencing. Phylogenetic analysis of the Venezuelan sequence allowed its classification in the clade IIb (Figure 2). 

### 3.3. Time-Course of the Early Outbreak in Latin America

On 31 August 2022, 7881 cases of monkeypox were reported in Latin America, which represented 15% of the 51,557 cases reported worldwide. Two countries reported more than 1000 cases at this date: Brazil (4693) and Peru (1463). The first countries in reporting cases were Argentina and Mexico on 3 June 2022, but it took more than two months to these countries to reach their first 100 cases: August 27 for Argentina and August 10 for Mexico. In Bolivia, Costa Rica, Cuba, Dominican Republic, Ecuador, Guatemala, Honduras, Panama, Paraguay, Uruguay, and Venezuela, less than 100 cases were detected in each country until 31 August 2022. In contrast, in Brazil and Peru, more than 200 cases were already detected after one month of the first case, and in Colombia, 200 cases were detected after two months [3]. 

## 4. Discussion

The algorithm developed by the INHRR proved to be useful for monitoring and detecting the first case of MPXV in Venezuela in June 2022. This assumes that the first cases in Venezuela were from individuals traveling and acquiring the infection abroad. However, as soon as the community transmission is suspected to occur, this algorithm may change to a direct MPXV qPCR detection in all samples.

MPXV sequences are classified into two clades: clade I, formerly known as the Central Africa clade, is the most pathogenic variant, with a mortality rate around 10%, and clade II (formerly known as the West African clade), which exhibits a significant lower pathogenicity [21,22,23,24,25,26]. Most of the sequences of the 2022 MPXV international outbreak are grouped into clade IIb, lineage B.1 [21], whereas some sequences from cases in the USA of 2022 belong to lineage A.1 [25]. The first Venezuelan isolate of MPXV was classified as clade II both by qPCR with the clade-specific primers and partial genome sequencing. Even if the complete genome sequence was not obtained, the information provided by 43% of the genome was sufficient to provide a satisfactory phylogenetic signal, though not enough for the lineage assignment. According to the travel history and date of this case, the sequence should be grouped within the lineage B.1. The divergence observed in the MPXV isolates during the course of the international outbreak has allowed the classification of this lineage into eight sub-lineages: B1.1 to B1.9 [26]. 

Although the first case of MPXV infection was detected relatively early in Venezuela, no further cases were detected until more than two months later. Only two additional cases were reported until the end of August, 2022: one on August 18 (returning from Brazil) and the other on August 23 (returning from Peru). As shown previously, Latin America has exhibited a heterogeneous MPXV epidemic pattern. Some countries exhibited an important increase in cases before 31 August 2022, whereas many others did not reach 100 reported cases at this date. Several factors might explain these different patterns: demographic parameters and international connectivity, for example. Additionally, an active surveillance system was not implemented in all the countries. Thus, significant underreporting might be expected in the region, also because of socio-cultural factors and the fear of stigmatization. 

In Peru, a new sub-lineage, B1.6, has emerged [26]. This can be the reflection of extensive community transmission in the country, more than in other Latin American countries. However, the apparent low dissemination of this international epidemic in some Latin American countries may change in the next months. Indeed, The Panamerican Health Organization classified, on 13 September 2022, the Americas region as a high risk of monkeypox [27].

## 5. Conclusions

The results stress the importance of active molecular surveillance for the control of the MPXV emergent outbreak. Although no further cases were detected in Venezuela until the end of August, when two more cases were detected, it cannot be ruled out that some cases might go unnoticed because of the fear of stigmatization, so the patient´s information should be managed discreetly by the epidemiological authorities.

## Figures and Tables

**Table 1:** Primers used for amplification of MPXV.

PCR	Primer	Sequence	Reference
qPCR (generic)	G2R_GF	5′-ggaaaatgtaaagacaacgaatacag-3´	[6]
	G2R_GR	5′-gctatcacataatctggaagcgta-3´	
	G2R_G probe	5′FAM-aagccgtaatctatgttgtctatcgtgtcc-3′BHQ1	
qPCR (Clade II)	G2R_WAF	5′-cacaccgtctcttccacaga-3´	[6]
	G2R_WAR	5′-gatacaggttaatttccacatcg-3´	
	G2R_WA probe	5′FAM-aacccgtcgtaaccagcaatacattt-3′BHQ1	
PCR (ORF F4L)	F4LF	5´cgttggaaaacgtgagtccgg-3´	[7]
Pox genus	F4LR	5´-attggcgttttttgcagccag-3´	
PCR (E5R)	E5RF	5´-atgttgatattaataatcgtattgtggtt-3´	[7]
	E5RR	5´-aaagtcaatacactcttaaagattctcaa-3´	
PCR (HA)	HAOUTF	5´-ccattggaaaaaacacagtac-3´	[8]
	HAOUTR	5´-ccaaatatattcccatagtc-3´	

## Data Availability

Nucleotide sequences of the partial complete genome have been deposited into the GISAID database (https://gisaid.org/) with the accession number EPI_ISL_15014548.
